# Supplementary material for: Nutritional status and TB treatment outcomes in Addis Ababa, Ethiopia: An ambi-directional cohort study
Source: PLoS One. 2021 Mar 2;16(3):e0247945. doi: 10.1371/journal.pone.0247945 (PMC7924797; doi:10.1371/journal.pone.0247945)
Supplement: S2 Fig — (DOCX) [file pone.0247945.s003.docx]

**S2 Fig**. Predicted probability of treatment successes by BMI at second months of treatment and sex among adult TB patients in public health centers of Addis Ababa, Ethiopia, 2019
