# Supplementary material for: Nutritional status and TB treatment outcomes in Addis Ababa, Ethiopia: An ambi-directional cohort study
Source: PLoS One. 2021 Mar 2;16(3):e0247945. doi: 10.1371/journal.pone.0247945 (PMC7924797; doi:10.1371/journal.pone.0247945)
Supplement: S1 Table — (DOCX) [file pone.0247945.s006.docx]

**S1 Table:** Univariable analysis of nutritional counselling and support association with change in body weight and BMI at the second months of treatment among adult TB patients in public health center of Addis Ababa, Ethiopia, 2019.

|  | **Unstandardized B coefficients** | **Standardized B coefficients** | **P-value** | **95% CI for B** |
| --- | --- | --- | --- | --- |
| **Body weight change at the second month of the treatment** | | | | |
| Nutritional counseling | .710 | 0.081 | 0.101 | -0.139, 1.56 |
| Food support | -.289 | -.025 | 0.618 | -1.427, 0.85 |
| **BMI change at the second month of the treatment** | | | | |
| Nutritional counseling | 0.172 | 0.049 | 0.324 | -.0171, 0.515 |
| Food support | -.221 | -0.047 | 0.345 | -0.680, 0.238 |

In univariable linear regression, we did not find a statistical association between nutritional counseling and food support and change in body weight (kg) and BMI at the second month of treatment.

**Nutritional counseling coded as**

0 NO

1 Yes (reference)

**Code for Food support coded as**

0 NO

1 Yes (reference)
