# Supplementary material for: Nutritional status and TB treatment outcomes in Addis Ababa, Ethiopia: An ambi-directional cohort study
Source: PLoS One. 2021 Mar 2;16(3):e0247945. doi: 10.1371/journal.pone.0247945 (PMC7924797; doi:10.1371/journal.pone.0247945)
Supplement: S2 Table — (DOCX) [file pone.0247945.s007.docx]

**S2 Table:** Univariable and multivariable analysis of BMI at the start of treatment association with treatment outcomes among adult TB patients in public health center of Addis Ababa, Ethiopia, 2019.

| Explanatory Variables | Successful treatment outcomes | Unsuccessful treatment outcomes | UAOR | 95% CI | AOR | 95% CI |
| --- | --- | --- | --- | --- | --- | --- |
| **BMI at the start of treatment** |  |  |  |  |  |  |
| BMI≥18.5 kg/m^2^ | 254 | 18 | 2.42** | 1.29, 4.55 | 2.15** | 1.05, 4.39 |
| BMI<18.5 kg/m^2^* | 157 | 27 |  |  |  |  |
| **Sex** |  |  |  |  |  |  |
| Male | 217 | 34 | 0.36** | 0.17, 0.73 | 0.36** | 0.16, 0.78 |
| Female * | 194 | 11 |  |  |  |  |
| Age |  |  | 0.93** | 0.91, 0.95 | 0.93** | 0.91,0.96 |
| **TB-HIV co-infection** |  |  |  |  |  |  |
| Yes | 64 | 17 | 0.30** | 0.15, 0.58 | 0.51 | 0.23, 1.10 |
| No* | 347 | 28 |  |  |  |  |
| **Type of TB and AFB sputum smear positivity** |  |  |  |  |  |  |
| Smear positive pulmonary TB | 188 | 19 | 1.15 | 0.61, 2.15 | 0.64 | 0.23, 1.73 |
| Smear negative pulmonary TB | 104 | 19 | 0.46** | 0.24, 0.87 | 0.46 | 0.17, 1.25 |
| Extra pulmonary TB* | 119 | 7 |  |  |  |  |

*referent, ** statistically significant association with P-value <0.05, UAOR-Unadjusted odds ratio, AOR-Adjusted odds ratio, CI- Confidence interval
