# Supplementary material for: Nutritional status and TB treatment outcomes in Addis Ababa, Ethiopia: An ambi-directional cohort study
Source: PLoS One. 2021 Mar 2;16(3):e0247945. doi: 10.1371/journal.pone.0247945 (PMC7924797; doi:10.1371/journal.pone.0247945)
Supplement: S3 Table — (DOCX) [file pone.0247945.s008.docx]

**S3 Table**: Univariable and multivariable analysis of BMI in the second month of treatment association with treatment outcomes among adult TB patients in public health center of Addis Ababa, Ethiopia, 2019.

| Explanatory Variables | Successful treatment outcomes | Unsuccessful treatment outcomes | UAOR | 95% CI | AOR | 95% CI |
| --- | --- | --- | --- | --- | --- | --- |
| **BMI after 2 months of treatment** |  |  |  |  |  |  |
| BMI≥18.5 kg/m^2^ | 282 | 8 | 3.55** | 1.43, 8.78 | 3.55** | 1.29, 9.73 |
| BMI<18.5 kg/m^2^* | 129 | 13 |  |  |  |  |
| **Sex** |  |  |  |  |  |  |
| Male | 217 | 34 | 0.36** | 0.17,0.73 | 0.13** | 0.02, 0.59 |
| Female | 194 | 11 |  |  |  |  |
| Age |  |  | 0.93** | 0.91 ,0.95 | 0.95** | 0.92, 0.98 |
| **TB-HIV co-infection** |  |  |  |  |  |  |
| Yes | 64 | 17 | 0.30** | 0.15, 0.58 | 0.41 | 0.14, 1.18 |
| No* | 347 | 28 |  |  |  |  |
| **Type of TB and AFB sputum smear positivity** |  |  |  |  |  |  |
| Smear positive pulmonary TB | 188 | 19 | 1.15 | 0.61, 2.15 | 1.21 | 0.35, 4.0 |
| Smear negative pulmonary TB | 104 | 19 | 0.46** | 0.24, 0.87 | 1.07 | 0.28, 4.0 |
| Extra pulmonary TB* | 119 | 7 |  |  |  |  |

*referent, ** statistically significant association with P-value <0.05, UAOR-Unadjusted odds ratio, AOR-Adjusted odds ratio, CI- Confidence interval
