# Supplementary material for: Nutritional status and TB treatment outcomes in Addis Ababa, Ethiopia: An ambi-directional cohort study
Source: PLoS One. 2021 Mar 2;16(3):e0247945. doi: 10.1371/journal.pone.0247945 (PMC7924797; doi:10.1371/journal.pone.0247945)
Supplement: S1 File — (PDF) [file pone.0247945.s009.pdf]

English version Questionnaire

Identification		
Sub city name : __________________	Health facility name: : __________________	
ID number: ________________		
Date of interview ________________	Time started____________	Time finished__________
Data collector's Name _________________	signature ________________	Date _________________
Supervisor's Name __________________	signature ________________	Date __________________

Section 1: Socio-Demographic characteristics Information

S.No.	QEUSTION	Response	Code
101	Your sex	1= Male 2=Female	|___|
102	Your age (in years)		|___|
103	Your marital status	1=Married -<br>2=Living together<br>3= Divorced/separated<br>4=Widowed<br>5=Never Married and Living Together	|___|
104	Your educational status	1= No formal Education<br>2=1-8 Grade<br>3=9-10 Grade<br>4=11-12 Grade<br>5= Above 12 Grade	|___|
105	Your occupation	1=Governmental employee<br>2= Daily laborer<br>3= Merchant<br>4=House wife<br>5=Student<br>Other (specify------------------)	|___|

Section 2: Clinical characteristics information

S.NO	Question	Response	Code
201	AFB sputum smear positivity	1=Smear positive pulmonary TB<br>2=Smear Negative pulmonary TB<br>3=Extra Pulmonary TB	|___|
202	TB HIV Co-infection	1=Yes<br>2=No	|___|
203	Other Non-communicable diseases	1=Diabetes<br>2=Cancer<br>3=Hypertension<br>4=Other specify	|___|
204	History of Tuberculosis	1=New case<br>2= Relapsed case<br>3= Treatment failure	|___|
205	Initial date of treatment (DD-MM-YYYY)	|___||___||___||___|||___|___||___||___|	|___|

Section 3: Nutritional status assessment

S.N	Nutritional measurement	At the start of treatment	At two months of treatment	At end of six months of treatment
301	Bodyweight in kilogram (Kg)			
302	Height in centimeter(cm)			
303	BMI (kg/h^2)			

Section 4: Nutritional Counseling and Support

S.N	Question	Response	Code
401	Have you got nutritional counseling from a health care provider in this facility?	1. Yes<br>2. No	|___|
402	Have you got food support from any source?	1. Yes<br>2. No	|___|

Section 5: TB treatment outcomes at end of 6 months

S.N	Status of the Treatment	Response	Code
501	Treatment Completed	1=Yes<br>2=No	|___|
502	Treatment cured	1=Yes<br>2=No	|___|
503	Treatment success	1=Yes<br>2=No	|___|
504	Default	1=Yes<br>2=No	|___|
505	Death	1=Yes<br>2=No	|___|
506	Date of Outcome DD/MM/YYY	|___||___||___||___|||___|___||___||___|	|___|

Section 6: Smear sputum test result only for pulmonary TB Patient only

S.N	Smear sputum test	Response	Code
601	At end of two months of treatment	1=Positive<br>2=Negative	|___|
602	At end of the fifth month of treatment	1=Positive<br>2=Negative	|___|
603	At end of six months of treatment	1=Positive<br>2=Negative	|___|
