# Supplementary material for: Nutritional status and TB treatment outcomes in Addis Ababa, Ethiopia: An ambi-directional cohort study
Source: PLoS One. 2021 Mar 2;16(3):e0247945. doi: 10.1371/journal.pone.0247945 (PMC7924797; doi:10.1371/journal.pone.0247945)
Supplement: S2 File — (PDF) [file pone.0247945.s010.pdf]

የአማርኛ የመጠየቅያ ቅፅ

የክፍለከተማ ስም፡_____________________ የጤና ድርጅቱ ስም፡_____________________.
የመጠይቁ መለያ ቁጥር፡__________________ የታካሚው የካርድ ቁጥር፡__________________.
ሕክምናውን የተጀመረበት ቀን፡________________የሚያለቅበት ቀን ሰዓት፡______________________.
መረጃ የተሰበሰበት ቀን፡__________________________.
የመረጃ ሰብሳቢው ስም፡____________ፊርማ፡________ቀን፡____________________.
ተቆጣጣሪው ስም፡___________ፊርማ፡__________ ቀን፡_______________________.

ክፍል 1፡ ስለ ቤተሰብ አጠቃላይ የመረጃ ሁኔታ መጠይቅ

ተ.ቁ	ጥያቄ	መልስ	ከድ
101	ፆታ	1= ወንድ<br>2= ሴት	|___|
102	እድሜ		|___|
103	የጋብቻ ሁኔታ	1= ያገባ<br>2= የተለያየ<br>3= የሞተበት<br>4= ያላገባ	|___|
104	የትምህርት ሁኔታ	1= ማንበብ እና መፃፍ የሚችል<br>2= ከ1-8 ክፍል<br>3= ከ 9 – 10 ክፍል<br>4= የቀለም/የሞያ ት/ት ከ 11–12 ክፍል<br>5= ከ12 ክፍል በላይ	|___|
105	ሥራ	1= የመንግስት ሰራተኛ<br>2= የቀን ሰራተኛ<br>3= ነጋዴ<br>4= የቤት እመቤት<br>5= ተማሪ<br>6= ሌላ ከሆን ይጥቀሱ	|___|

ክፍል 2፡ ከበሽታ ጋር የተገናኙ መጠይቆች

ተ.ቁ	ጥያቄ	መልስ	ኮድ
201	የቲቢ አይነት	1= የአክታ ፖዘቲቭ የሳንባ ቲቢ<br>2= የአክታ ኔጋቲቭ የሳንባ ቲቢ<br>3= ከሳን ባውጪ ቲቢ	|___|
202	ቲቢ/ኤችአይቪ ህምተኛ ኖት	1= አዎ<br>2= አይደለም	|___|
203	ሌላ ተላላፊ ያልሆኑ በሽታዎች አለቦት	1= የስኳር ብሽታ<br>2= ካንሰር<br>3= የደም ግፊት<br>4= ሌላ ካለ ይጥቀሱ	|___|
204	የቲቢ ሁኔታ	1= አዲስ ቲቢ<br>2= ሕክምናው ያልሰራ<br>3= ያገረሸ<br>4= ያቋረጠ	|___|
205	ህክምና የተጀመረበት ቀን<br>(ቀን/ወር/ዓመት)	|___||___||___||___|||___|___||___||___	

ክፍል 3፡ ስነ ምግብ ሁኔታ መዳሰስ

**ተ.ቁ**	**የስነ ምግብ ሁኔታ**	**ሕክምና ሲጀምሩ**	**ሁለተኛ ወር ሲጨርሱ**	**ሕክምናውን ሲጨርሱ**	**ኮድ**
301	የሰውነት ክበደት በኪሎ				|___|
302	የሰውነት ቁመት በሴንቲሜትር				|___|
303	ቢምይ (BMI)				|___|

ክፍል 4፡ የስነ ምግበ የምክርና የድጋፍ አገልግሎት ዳሰሳ

**ተ.ቁ**	**መጠይቅ**	**መልስ**	**ኮድ**
401	የስነ ምግበ የምክር አገልግሎት ከዚህ የጤና ድርጀት ያገኛሉ	1= አዎ<br>2= አይደለም	|___|
402	የተጨማረ ምግብ ወይም የምግብ ድጋፍ ከዚህ ወይም ከሌላ ድርጀት አግኝተው ያውቃሉ	1= አዎ<br>2= አይደለም	|___|

ክፍለ 5፡ የቲቢ ህክምነና ውጤት በስድስተኛ ወር መጨረሻ

**ተ.ቁ**	**501**	**502**	**503**	**504**	**505**
**የቲቢ ህክምነና ውጤት**	**ሕክምና የጨረሰ**	**ሕክምና የተሳካ**	**በሕክምና የዳነ**	**ያቋረጠ**	**የሞተ**
**መልስ**	1= አዎ<br>2= አይደለም	1= አዎ<br>2= አይደለም	1= አዎ<br>2= አይደለም	1= አዎ<br>2= አይደለም	1= አዎ<br>2= አይደለም
**ኮድ**	|___|	|___|	|___|	|___|	|___|

ከመተ የሞተበት ቀን ወር ዓም

ክፍል 6፡ የአክታ ምርመራ ውጤት (ለሳንባ ቲቢ)

**ተ.ቁ**	**601**	**602**	**603**	**604**
**የቲቢ ህክምነና ወር**	**ሁለተኛ ወር ሲጨርሱ**	**በአመስተኛ ወር**	**በስድስተኛ ወር መጨረሻ**	**ያልተሳካ ሕክምና**
**የአክታ ውጤት**	1= ፖዘቲቭ<br>2= ኔጋቲቭ	1= ፖዘቲቭ<br>2= ኔጋቲቭ	1= ፖዘቲቭ<br>2= ኔጋቲቭ	1= አዎ<br>2= አይደለም
**ኮድ**	|___|	|___|	|___|	|___|
