# Supplementary material for: Nutritional status and TB treatment outcomes in Addis Ababa, Ethiopia: An ambi-directional cohort study
Source: PLoS One. 2021 Mar 2;16(3):e0247945. doi: 10.1371/journal.pone.0247945 (PMC7924797; doi:10.1371/journal.pone.0247945)
Supplement: Attachment — Submitted filename: Reviewer.docx [file pone.0247945.s011.docx]

**Reviewer’s comments**

- Sample size was based on reference 24 . It has been stated hat it is based on Indian study ‘Kindly check the reference
- Patients in the exposed group have larger number of smear positive cases . Has the correlation with outcomes seen in relation with smear positivity. Has it been considered in adjusted odds ratio.
- Death has been reported to occur in lesser days in unexposed group as compared to exposed group. What is the likely cause . Kindly discuss.
- Kindly check the table 2 . Kindly check outcome numbers for BMI at 2 months .
- Nutritional status has been compared with treatment outcome in various studies. What is the novelty of this study. While BMI is significantly associated with outcome, weight is not associated . kindly explain.
- What was the change in weight seen in 2 months and 6 months. kindly mention the numbers.
- Grammatical errors needs to be corrected.
