# Supplementary material for: Nutritional status and TB treatment outcomes in Addis Ababa, Ethiopia: An ambi-directional cohort study
Source: PLoS One. 2021 Mar 2;16(3):e0247945. doi: 10.1371/journal.pone.0247945 (PMC7924797; doi:10.1371/journal.pone.0247945)
Supplement: Attachment — Submitted filename: Response to Reviewers.docx [file pone.0247945.s012.docx]

**Response to reviewer’s comments**

PONE-D-20-29191
Nutritional status and TB treatment outcomes in Addis Ababa, Ethiopia: An ambi-directional cohort study

PLOS ONE

Dear editor and reviwers

We would like to thank you for your comments and suggestions you are given for the above manuscript. We have considered all the comments one by one and give you the following response.

1. Please ensure that your manuscript meets PLOS ONE's style requirements, including those for file naming. The PLOS ONE style templates can be found at

<https://journals.plos.org/plosone/s/file?id=wjVg/PLOSOne_formatting_sample_main_body.pdf> and

<https://journals.plos.org/plosone/s/file?id=ba62/PLOSOne_formatting_sample_title_authors_affiliations.pdf>

**Response**: We prepared the new version manuscript according to the PLOS ONE style requirement.

2. Please clearly state all the variables you included in the multivariable regression model.

**Response**: All variables included in the multivariable regression model are clearly stated in the new version of the manuscript document in results section paragraph 5. Sex, age, TB/HIV co-infection, type of TB and AFB sputum smear positivity were all included in the multivariable model.

3. In statistical methods, please clarify whether you corrected for multiple comparisons.

**Response**: P-values in this analysis were not adjusted to account for multiple comparisons. This information is included in the statistical methods section of the revised manuscript.

4. Please include additional information regarding the survey or questionnaire used in the study and ensure that you have provided sufficient details that others could replicate the analyses. For instance, if you developed a questionnaire as part of this study and it is not under a copyright more restrictive than CC-BY, please include a copy, in both the original language and English, as Supporting Information, or include a citation if it has been published previously.

**Response:** We prepared the questionnaire for this study using WHO guidelines and similar studies. We provided additional information about the questionnaire in the data collection methods. Both the English and Amharic versions of the study questionnaire are attached as supporting information.

5. As part of your revision, please complete and submit a copy of the RECORD checklist, a document that aims to improve reporting and reproducibility of observational studies that use routinely-collected data for purposes of post-publication data analysis and reproducibility: ([http://record-statement.org](http://record-statement.org/)). Please include your completed checklist as a Supporting Information file. Note that if your paper is accepted for publication, this checklist will be published as part of your article.

**Response**: We completed the RECORD checklist and attached it as supporting information.

6.We suggest you thoroughly copyedit your manuscript for language usage, spelling, and grammar. If you do not know anyone who can help you do this, you may wish to consider employing a professional scientific editing service.  

Whilst you may use any professional scientific editing service of your choice, PLOS has partnered with both American Journal Experts (AJE) and Editage to provide discounted services to PLOS authors. Both organizations have experience helping authors meet PLOS guidelines and can provide language editing, translation, manuscript formatting, and figure formatting to ensure your manuscript meets our submission guidelines. To take advantage of our partnership with AJE, visit the AJE website (http://learn.aje.com/plos/) for a 15% discount off AJE services. To take advantage of our partnership with Editage, visit the Editage website ([www.editage.com](http://www.editage.com/)) and enter referral code PLOSEDIT for a 15% discount off Editage services.  If the PLOS editorial team finds any language issues in text that either AJE or Editage has edited, the service provider will re-edit the text for free.

 Upon resubmission, please provide the following:

- The name of the colleague or the details of the professional service that edited your manuscript
- A copy of your manuscript showing your changes by either highlighting them or using track changes (uploaded as a *supporting information* file)
- A clean copy of the edited manuscript (uploaded as the new *manuscript* file)

**Response**: All grammatical errors have been corrected in the new version manuscript with a professional English language editor. The copy of the English edited manuscript showing tracked changes is attached as a “supporting information” file and the clean copy of the edited manuscript is attached as a “manuscript” file.

7.We note that you have indicated that data from this study are available upon request. PLOS only allows data to be available upon request if there are legal or ethical restrictions on sharing data publicly. For information on unacceptable data access restrictions, please see <http://journals.plos.org/plosone/s/data-availability#loc-unacceptable-data-access-restrictions>.

 In your revised cover letter, please address the following prompts:

 a) If there are ethical or legal restrictions on sharing a de-identified data set, please explain them in detail (e.g., data contain potentially identifying or sensitive patient information) and who has imposed them (e.g., an ethics committee). Please also provide contact information for a data access committee, ethics committee, or other institutional body to which data requests may be sent.

 b) If there are no restrictions, please upload the minimal anonymized data set necessary to replicate your study findings as either Supporting Information files or to a stable, public repository and provide us with the relevant URLs, DOIs, or accession numbers. Please see <http://www.bmj.com/content/340/bmj.c181.long> for guidelines on how to de-identify and prepare clinical data for publication. For a list of acceptable repositories, please see <http://journals.plos.org/plosone/s/data-availability#loc-recommended-repositories>.

 We will update your Data Availability statement on your behalf to reflect the information you provide.

**Response**: Thank you for asking for clarification regarding data availability. We are unable to fully share the data due to identifiable information such as examination date, death date, and default date. The data were collected from health facilities in Addis Ababa city and have information such as the health facility name, sex, and age of the participants. We included all information related to the research questions in the manuscript document. We confirm that the data available in the manuscript document is minimum data set.

 8.Thank you for stating the following in the Acknowledgments Section of your manuscript:

[This work was supported in part by the NIH/Fogarty International Center Global Infectious Disease 341 grant D43TW009127.]

We note that you have provided funding information that is not currently declared in your Funding Statement. However, funding information should not appear in the Acknowledgments section or other areas of your manuscript. We will only publish funding information present in the Funding Statement section of the online submission form.

Please remove any funding-related text from the manuscript and let us know how you would like to update your Funding Statement. Currently, your Funding Statement reads as follows:

 [This research work was funded by Addis Ababa University thematic research project fund.  ]

 Please include your amended statements within your cover letter; we will change the online submission form on your behalf.

**Response**: We removed any funding related text from the manuscript document as suggested. We would like to update the funding statement to include [This research work was funded by Addis Ababa University thematic research project fund and supported in part by the NIH/Fogarty International Center Global Infectious Disease 341 grant D43TW009127.]. This funding statement amendment request is included in the cover letter.

 9. Please ensure that you refer to Figure 3 in your text as, if accepted, production will need this reference to link the reader to the figure.

**Response**: Figure 3 is now cited in the text in the results section paragraph 6 of the revised manuscript.

 10. Please include captions for your Supporting Information files at the end of your manuscript, and update anyin-text citations to match accordingly. Please see our Supporting Information guidelines for more information: <http://journals.plos.org/plosone/s/supporting-information>.

**Response**: Captions for supporting information have been included at the end of the manuscript.

Reviewers' comments:

Reviewer's Responses to Questions

**Comments to the Author**

1. Is the manuscript technically sound, and do the data support the conclusions?

The manuscript must describe a technically sound piece of scientific research with data that supports the conclusions. Experiments must have been conducted rigorously, with appropriate controls, replication, and sample sizes. The conclusions must be drawn appropriately based on the data presented.

Reviewer #1: Partly

Reviewer #2: Partly

2. Has the statistical analysis been performed appropriately and rigorously?

Reviewer #1: I Don't Know

Reviewer #2: Yes

3. Have the authors made all data underlying the findings in their manuscript fully available?

The [PLOS Data policy](http://www.plosone.org/static/policies.action#sharing) requires authors to make all data underlying the findings described in their manuscript fully available without restriction, with rare exception (please refer to the Data Availability Statement in the manuscript PDF file). The data should be provided as part of the manuscript or its supporting information, or deposited to a public repository. For example, in addition to summary statistics, the data points behind means, medians and variance measures should be available. If there are restrictions on publicly sharing data—e.g. participant privacy or use of data from a third party—those must be specified.

Reviewer #1: Yes

Reviewer #2: Yes

4. Is the manuscript presented in an intelligible fashion and written in standard English?

PLOS ONE does not copyedit accepted manuscripts, so the language in submitted articles must be clear, correct, and unambiguous. Any typographical or grammatical errors should be corrected at revision, so please note any specific errors here.

Reviewer #1: Yes

Reviewer #2: Yes

5. Review Comments to the Author

Please use the space provided to explain your answers to the questions above. You may also include additional comments for the author, including concerns about dual publication, research ethics, or publication ethics. (Please upload your review as an attachment if it exceeds 20,000 characters)

Reviewer #1: Sahile Z and co-workers performed a retrospective and prospective cohort study of 456 TB patients in Addis Ababa, Ethiopia, comparing the TB treatment outcomes in persons with low BMI (<18.5 kg/m^2^) vs. those with BMI ≥ 18.5 kg/m^2^ at treatment initiation and after 2 months of treatment. Treatment outcomes were classified as “successful” – based on 1) negative AFB sputum smear at end of treatment + at least one other previous test or 2) completed treatment with resolution of symptoms. Unsuccessful outcome was defined as those with persistently positive AFB sputum smear at 5 months or later of TB treatment, died, or defaulted from treatment.

Comments:
1. How was the diagnosis of TB (pulmonary or extrapulmonary) made? Based on their aforementioned definition for “successful” and “unsuccessful” outcomes, it seems as if only AFB smear was done. If so, how was the diagnosis made for the smear-negative TB (which made up of ~26-27% of the TB subjects) and of the extrapulmonary TB (14-37% of the subjects)? How do they know that some of the acid-fast bacteria may be non-tuberculous mycobacteria. I think this should at least be mentioned as a limitation of the study.

**Response**: We collected information on TB diagnosis, BMI and treatment outcomes from medical records. A diagnosis of TB disease was made according to the Ethiopia National Tuberculosis Guidelines. TB cases were considered confirmed if they had a positive result for AFB sputum smear, Xpert MTB/RIF, or *Mycobacterium tuberculosis* culture. TB cases diagnosed clinically by an experienced clinician were also included. However, the reviewer correctly points out that most cases were diagnosed using AFB sputum smear. Given many TB cases did not have microbiologic confirmation, we have added this to the limitation section at the end of the manuscript.

2. The authors referred to the low BMI group as “exposed” and the normal/high BMI group as “unexposed.” What is the reasoning behind this? This seems unnecessary and actually makes the manuscript more difficult to understand. Indeed, in the last paragraph of the Results and first paragraph of the Discussion, they used the terms underweight and normal/overweight, which is less ambiguous than re-naming the groups to terms (exposed and unexposed) that are cryptic. Alternative, they could refer to the groups as low BMI or normal/high BMI rather than exposed and unexposed.

**Response**: In the revised manuscript, we exclusively refer to the study groups as “underweight” and “normal and overweight” to avoid confusion.

3. The comparisons in Table 1 between the underweight and normal/overweight patients need P values to determine significant differences (or lack thereof). While relatively smaller in numbers, the underweight group had higher number of prior relapsed TB and greater number who were infected with HIV, which could be confounders for worse treatment outcomes. We also know that failed prior therapy (relapsed) is likely associated with worse prognosis as it is a substrate for resistance to greater number of anti-TB drugs. Also, do you have data on number of prior treatments in the “relapsed” groups?

**Response:** In the revised manuscript, we used a chi-square to assess differences in demographic and clinical factors between underweight and normal/overweight groups. P-values have been added to tables 1 and 2 as suggested by the reviewer. We assessed the association between potential confounding factors such as sex, age, TB/HIV co-infection, and Type of TB and AFB sputum smear positivity category in the multivariable analysis. Tables S1 and S2 of the revised manuscript contain the point estimate, 95% CI and p-value of both the univariable and multivariable logistic regression analysis for each variable. Unfortunately we were unable to obtain data on the number of prior treatments received in persons previously treated for TB disease.

4. Similarly, the comparisons in Table 2 between the underweight and normal/overweight patients need P values to determine significant differences (or lack thereof). The number of HIV positive cases was ~1.7X greater in the underweight group vs the normal/overweight group and certainly could impact outcome. What % of those who are HIV positive were receiving anti-retroviral therapy as this may obviously impact outcome in several ways including improving host immunity and adverse interactions of anti-HIV drugs with anti-TB drugs.

**Response:** As suggested by the reviewer, we used a chi-square test to assess the association between clinical factors and BMI P-values were added to Table 2 of the revised manuscript. As above, the point estimate, 95% CI and p-value for each co-variate as calculated using univariable and multivariable logistic regression is provided in tables S1 and S2. While the proportion of persons with HIV on effective antiretroviral therapy is relevant, this information was not provided in the medical record. We have added this as a limitation in the revised manusceript.

5. In the abstract, the first sentence of Results section contradicts the first sentence of the Conclusion section. That is, I think the first sentence of the Conclusion should be “….underweight at baseline and after 2 months of treatment was a predictor for treatment failure” (rather than treatment success as it is written).

**Response**: The reviewer correctly points out that being underweight at baseline and after 2 months of treatment was a predictor for an unsuccessful treatment outcome. This change was made in the revised manuscript.

6. Are there data on the number of TB patients in each of the successful and unsuccessful treatment outcomes with MTB isolates that are rifampin resistant, MDR-TB, or XDR-TB? It seems likely that high levels of drug resistance may actually play a bigger role on whether the outcome is successful or unsuccessful. Along that line, were any patients diagnosed with Cepheid GeneXpert or another nucleic acid amplification test, which may confirm or refute the diagnosis of MDR-TB and thus one can compare patients with drug-susceptible TB with each other and those with MDR-TB with each other.

**Response**: The reviewer correctly points out that most TB diagnoses were based on AFB sputum smear and/or assessment of the treating clinician. As a result, the impact of drug resistance on treatment outcomes cannot be fully ascertained by this analysis. We have added this limitation to the limitation section at the end of the revised manuscript.

7. What was the general treatment regimen used and was it different between the underweight vs the normal/overweight groups? Those who were underweight were more likely to be smear-positive…was cavitary disease more common in this group since it may be a marker for more severe disease as well as greater bacterial burden.

**Response:** In this study, we included TB patients who were treated with six months of anti-TB therapy. The treatment regimen included two months of rifampicin, isoniazid, pyrazinamide, and ethambutol followed by four months of rifampicin and isoniazid and was the same for both the underweight and normal/overweight patients. This information was added to the revised methods section. Chest radiographs were not routinely performed so data is not available on the presence or absence of cavitary pulmonary disease.

8. In line 206, they noted “Among 106 pulmonary TB patients in the exposed group…” I am unclear where this “106” number is derived from. It is not in Table 2 I think.

**Response:** We agree with the reviewer that this point requires clarificaiton. Among 110 underweight pulmonary TB patients and 97 normal or overweight pulmonary TB patients, there were only 106 in the underweight group and 90 in the normal and overweight group who completed the 6-month treatment period. The lower numbers are due to death and treatment default over the course of the study. We have clarified this point in the revised manuscript.

9. In Table 3, since the UAOR for the weight change for 2 and 6 months after treatment, shouldn’t you have the data for the mean weight changes for the successful and unsuccessful treatment groups for these times after treatment was started? I think it would be important to show to the readers what directionality (weight gain, weight loss) the weight change was going.

**Response**: We included the mean weight change after the second and sixth months of the treatment with the standard deviation in Table 3 in the revised manuscript as suggested by the reviewer.

10. In the first paragraph of the Discussion, the text goes back and forth unsuccessful and successful outcome. I think it would be less confusing to use one or the other whenever possible and not a combination of both. For example, one could say greater successful outcome vs. lower successful outcome, rather than greater successful outcome vs. higher unsuccessful outcome.

**Response**: We agree with the reviewer that this approach created confusion. We have edited the revised manuscript so the first paragraph of the discussion focuses on associations with a successful treatment outcome.

11. In the fifth paragraph of the Discussion, they noted “…we did not find any association between treatment outcome and weight at any time point….” which I do not understand. First of all, they examined BMI and not weight. Second, I thought they DID find an association between low BMI at baseline and at 2 months and lower successful outcomes. They also noted that “This is consistent with a Peru study that found baseline weight was not associated with treatment outcomes.” I don’t understand how this is consistent with the Peru study since low baseline BMI in their study was associated with lower treatment success; i.e., while I acknowledge the Peru study examined weight and the authors here examined BMI, it seems to me that their data, if anything, is NOT consistent with the Peru study.

**Response**: The reviewer correctly states that the primary objective of our study was to examine the association between BMI and treatment outcome. Our secondary objective was to assess the association between treatment outcomes and body weight not adjusted for height at diagnosis and after two months and six months of TB treatment as well as bodyweight change after two months and six months of treatment with treatment outcome. Thus, our findings related to bodyweight not adjusted for height do differ from the Peruvian and Iranian studies. We have added additional language to the revised manuscript to clarify when discussing bodyweight not adjusted for height.

12. In their Conclusion paragraph, the first sentence is ambiguous and needs more description of directionality; e.g., I think it is better phrased as “Low BMI at start of treatment was a proxy indicator for worse TB treatment outcomes while low BMI after 2 months of treatment was a strong predictor for unsuccessful treatment outcome.

**Response**: We have made this correction in the revised manuscript as suggested by the reviewer.

**Reviewer #2:**

1. Sample size was based on reference 24 . It has been stated that it is based on Indian study ‘Kindly check the reference

**Response**: The reviewer correctly points out that there was inconsistency between the reference citation and the setting of the study upon which the sample size calculation was based. The study was based in Myanmar and Zimbabwe. We corrected the revised manuscript accordingly.

2. Patients in the exposed group have a larger number of smear-positive cases. Has the correlation with outcomes seen in relation to smear positivity. Has it been considered in adjusted odds ratio.

**Response**: The reviewer correctly points out that a significantly larger number of AFB smear-positive participants were in the underweight group ((P<0.05). Thus, a variable for AFB-smear positive status was included in the multivariable analysis as indicated in supplementary tables 1 and 2.

3. Death has been reported to occur in lesser days in unexposed group as compared to exposed group. What is the likely cause . Kindly discuss.

**Response**: The reviewer correctly indicates that the median days to death was lower among normal or overweight TB patients compared to underweight patients. We have added further discussion on this point in the discussion section paragraph 4 of the revised manuscript.

4. Kindly check the table 2 . Kindly check outcome numbers for BMI at 2 months .

**Response:** After rechecking table 2 for the inconsistency with outcome numbers we did not identify any errors. It should be noted that patients who default and or die before two months of treatment were excluded from this analysis because BMI after the second month of treatment could not be measured.

5. Nutritional status has been compared with treatment outcome in various studies. What is the novelty of this study? While BMI is significantly associated with outcome, weight is not associated. kindly explain.

**Response:** While studies in other countries have demonstrated an association between BMI and adverse TB treatment outcomes, this is the first study, to our knowledge, that examines whether BMI predicts TB treatment outcomes in Ethiopia. Given the wide heterogeneity in the host response to TB disease, it is important that clinical factors predictive of adverse treatment outcomes be validated in populations with diverse genetic backgrounds. Thus, it is important to also examine the association between nutritional status and TB treatment outcomes in the Ethiopian context.

The reviewer correctly points out that while low BMI at baseline and after 2 months of treatment was associated with unsuccessful treatment outcomes, bodyweight not adjusted for height was not. We believe this occurred because unadjusted bodyweight is not an appropriate measure of nutritional status. We have added additional language to the revised manuscript to clarify when we are referring to measures of bodyweight not adjusted for height.

6. What was the change in weight seen in 2 months and 6 months. kindly mention the numbers.

**Response**: In the revised manuscript, we included the mean weight change after the second and sixth months of treatment with the standard deviation in Table 3 as suggested by the reviewer.

7. Grammatical errors needs to be corrected.

**Response**: We have corrected grammatical mistakes in the revised manuscript with an editor proficient in English.

6. PLOS authors have the option to publish the peer review history of their article ([what does this mean?](https://journals.plos.org/plosone/s/editorial-and-peer-review-process#loc-peer-review-history)). If published, this will include your full peer review and any attached files.


If you choose “no”, your identity will remain anonymous but your review may still be made public.


**Do you want your identity to be public for this peer review?** For information about this choice, including consent withdrawal, please see our [Privacy Policy](https://www.plos.org/privacy-policy).

Reviewer #1: **Yes:** Edward Chan

Reviewer #2: No
