# Supplementary material for: Nutritional status and TB treatment outcomes in Addis Ababa, Ethiopia: An ambi-directional cohort study
Source: PLoS One. 2021 Mar 2;16(3):e0247945. doi: 10.1371/journal.pone.0247945 (PMC7924797; doi:10.1371/journal.pone.0247945)
Supplement: Attachment — Submitted filename: Reviewer.docx [file pone.0247945.s013.docx]

Reviewer’s comments:

Kindly mention the details of nutritional counselling and nutrition support and how were they provided

Kindly mention relation of nutritional support and nutritional counselling to BMI at 2 month and weight change
