# Supplementary material for: Nutritional status and TB treatment outcomes in Addis Ababa, Ethiopia: An ambi-directional cohort study
Source: PLoS One. 2021 Mar 2;16(3):e0247945. doi: 10.1371/journal.pone.0247945 (PMC7924797; doi:10.1371/journal.pone.0247945)
Supplement: Attachment — Submitted filename: Response to Reviewers.docx [file pone.0247945.s014.docx]

Response to Reviewers’ comments:

PONE-D-20-29191R1

Nutritional status and TB treatment outcomes in Addis Ababa, Ethiopia: An ambi-directional cohort study PLOS ONE

Dear Reviewer

We would like to thank you for your comments and suggestions you are given for the above manuscript. We have considered your comments and give you the following response.

1. Kindly mention the details of nutritional counselling and nutrition support and how were they provided

**Response:** In this study, nutritional counselling was assessed through self-report of patients for receiving nutritional related information from the health facility. Nutritional counselling was defined as receipt of any nutrition-related information or guidance by clinic staff. Individual nutrition support programs varied by study site and data on the content of each program was not collected. We have added this information to the revised methods section (Page 4, Line 136-138). This information is also mentioned in the discussion section in the last paragraph.

2. Kindly mention the relation of nutritional support and nutritional counselling to BMI at 2 months and weight change

**Response**: As per the reviewers’ suggestion we did a univariable linear regression analysis to assess the association between nutritional counselling and support with weight change and BMI change in the second month of treatment. We did not find a statistical association between nutritional counselling and support and change in weight and BMI in the second month of treatment. This information was added to the supplementary file (S1 Table).
